# Engineered FADD Induces Apoptosis via an Artificial Death-Inducing Signaling Complex (DISC)

**Published:** 2009-09

**Authors:** Emiko Suzuki, Tomoki Takashina, Manabu Nakayama

**Affiliations:** 1 *Department of Human Genome Research, Kazusa DNA Research Institute, 2−6−7 Kazusa-Kamatari, Kisarazu, Chiba, Japan*; 2 *Laboratory of Pharmacogenomics, Graduate School of Pharmaceutical Sciences, Chiba University, 2−6−7 Kazusa-Kamatari, Kisarazu, Chiba, Japan*

**Keywords:** apoptosis, FADD, biotechnology, DISC, engineered protein

## Abstract

An engineered Fas-associated death domain protein (FADD), 2DEDplusE—made previously by fusing the tandem DEDs of FADD to the E protein of lambda phage-greatly enhances apoptosis-inducing activity in adherent cells *in vitro*. To investigate the mechanism of apoptosis-inducing activity of this engineered FADD, we compared the apoptosis-inducing activity of various other engineered FADDs. The tandem DED of 2DEDplusE contributed most to the enhancement of apoptosis, and the E protein contributed moderately. The engineered factor produced artificial death-inducing signaling complex (DISC)-like signals in the cytoplasm that appear as grains under fluorescence microscopy. Membrane blebbing associated with apoptosis was observed just after formation of grain-like signals. Immunoprecipitation analysis demonstrated that 2DEDplusE-FLAG can bind p43/p41 forms of caspase 8 but E protein-FLAG cannot. Gel filtration analysis demonstrated that 2DEDplusE forms a large complex containing partially cleaved procaspase 8 (p43/p41) in the cytoplasm; the size of this complex varies greatly. In the absence of an extrinsic signal, the engineered FADD formed artificial DISC in the cytoplasm, and then its tandem DED activated procaspase 8, which in turn executed apoptosis. The engineered FADD complex closely mimicked intrinsic DISC and increased apoptosis-inducing activity.

## INTRODUCTION

Our previous paper reported that an engineered form of FADD (1, 2) greatly increases its apoptosis-inducing activity in both adherent NIH3T3 and HEK293 cells *in vitro* (3). The engineered FADD, named 2DEDplusE, was made by fusing the tandem DEDs of FADD to the E protein of lambda phage, a head coat protein with self-assembly activity. The apoptosis-inducing activity of 2DEDplusE is much stronger than that of unmodified FADD. Five hours after induction, approximately 90% of cells were affected and had detached. Approximately 24 hours after induction of 2DEDplusE expression, almost 100% of cells had died. We used the E protein of lambda phage because E protein has self-assembly activity in *Escherichia coli* (4, 5). We expected 2DEDplusE to have the same effect as DISC on the Fas ligand/Fas pathway, a well-characterized apoptosis pathway. We hypothesized that 2DEDplusE protein molecules produced in a cell assemble automatically as E protein self-associates, and then each DED binds procaspase 8. Next, assembled procaspase 8 is converted to the active form, which induces a strong apoptosis signal. This signal is based on the fact that procaspase 8 possesses weak proteinase activity even before it is processed and thus can self-activate to form caspase 8 (6). Although we believe that an artificial DISC or a 2DEDplusE complex that mimics DISC exists, it has yet to be demonstrated.

We also reported that apoptotic cells that were undergoing very early-stage dynamic membrane blebbing revive when exposed to another modified FADD with moderate apoptosis-inducing activity (7). The engineered FADD, named 2DED2DD, was produced by fusing tandem death effector domains (DEDs) and tandem death domains (DDs). Induction of apoptosis by 2DED2DD caused rapid blebbing. Eight hours after exposure to 2DED2DD most cells had shrunk, while some had detached from the flask surface. Twenty-four hours later, when activated caspase 3 had decreased, more than half the cells revived and appeared normal, probably due to the induction of unidentified anti-apoptotic proteins.

Determining the mechanism of the apoptosis-inducing activity of 2DEDplusE will not only unravel the workings of apoptosis and anti-apoptosis machinery *in vivo* but could also further improve this modified FADD system, for example, by making it a stronger apoptosis-inducing factor, even at very low expression levels. Elucidating the mechanism will also reveal the minimal essential part of this modified FADD (2DEDplusE) as well as will shed light on the regulation system and factors that control the strength of its apoptosis-inducing activity. The present study confirmed whether apoptotic cells harbor an artificial DISC of 2DEDplusE and whether the E protein of 2DEDplusE self-associates in eukaryotic cells. We also investigated why this modified FADD (2DEDplusE) has strong apoptosis-inducing activity.

## MATERIALS AND METHODS

### Construction of plasmids expressing modified FADD-tagged 3xFLAG

The plasmid pTREx-2DEDplusE allows production of a foreign protein under the control of a tetracycline repressor system and constitutively produces EGFP from a CMV promoter as described previously (3). pTREx-2DEDplusE-FLAG was constructed by inserting a 3xFLAG sequence into the C terminus of the 2DEDplusE open reading frame. pTREx-2DEDplusE-EGFP was constructed by deleting one of the CMV promoter regions from pTREx-2DEDplusE and fusing the C terminus of the 2DEDplusE open reading frame to the N terminus of EGFP. pTREx-E protein, pTREx-DEDplusE, pTREx-2DED, and pTREx-empty produce E protein, DEDplusE (a single DED plus E protein), 2DED, and nothing, respectively. All constructs were confirmed by DNA sequencing.

### Fluorescence microscopy analysis

Flp-in TREx 293 cells (Invitrogen) or HeLa cells were plated onto poly-D-lysine–coated glass bottom dishes (MatTek Corporation), and expression plasmids were transfected into the cells by using FuGene 6 Transfection Reagent (Roche). Typically, tetracycline induction was started soon after transient transfection or tetracycline was added 24 hours after subculture. Twenty-four hours after tetracycline induction, cells were washed with PBS, fixed with 4% paraformaldehyde in 0.1 M phosphate buffer and 3.4% sucrose for 30 min at room temperature. Subcellular localization of EGFP-fusion proteins was determined by examining the cells with an Olympus IX71 fluorescence microscope equipped with either UPlanApo 40×/0.85 or PlanApo 60×/1.40 oil immersion objectives. Observations were recorded with a Hamamatsu ORCA-ER CCD camera.

### Generation of stable expression cell lines

Flp-in TREx 293 cells (Invitrogen) were co-transfected with expression vector (pTREx-2DEDplusE-EGFP or pTREx-2DEDplusE-FLAG) and pOG44, which encodes Flp recombinase, using FuGene 6 (Roche). Because the Flp-in TREx 293 cells contain a single integrated FRT site, all of the hygromycin-resistant clones should be isogenic. After subculture of the stable cell line, we added tetracycline to a final concentration of 1 μg/ml and incubated the cells at 37°C in a CO_2_ incubator to induce expression of the gene of interest.

### Live cell imaging by time-lapse microscopy

Cells were observed using differential interference contrast microscopy (Olympus IX81) using a PlanApo N 60×/1.42 objective lens. The microscope was equipped with a CO_2_ incubator on a heated stage for live-cell microscopy (Olympus MI-IBC-I). Images were recorded using a CCD camera (Olympus DP30BW) and Lumina Vision software (Mitani Corp.). Typically, 5 × 10^4^ cells were plated onto poly-D-lysine-coated 35-mm glass bottom dishes. Twenty-four hours after transiently transfecting the cells, we added tetracycline to the culture medium. Images of the cells were recorded continually at 10 min intervals for 26 hours.

### Measuring cell death activity by transient transfection of original and modified FADD

The expression plasmids encoding unmodified FADD, modified FADD, or empty vector (control) were transfected into Flp-in TREx 293 cells (Invitrogen) using FuGene 6 Transfection Reagent (Roche). Cells were plated at a density of 2 × 10^5^ cells per dish with appropriate DMEM medium on poly-D-lysine-coated 35-mm glass bottom dishes (MatTek Corporation), then tetracycline was added. Twenty-four hours after transfection and tetracycline induction, EGFP signal was recorded by means of a fluorescence microscope (Olympus IX81) with a UPlanFL N 20×/0.50 lens. At least nine fields of cells per dish (cells were unfixed) were recorded using a CCD camera (Olympus DP30BW) (typically 100 msec). The percentage of apoptotic cells was determined by counting at least 300 green cells (n ≥ 3), basically according to a procedure described previously (8). Briefly, round green cells and/or green cells exhibiting plasma membrane blebbing and cell shrinkage were scored as being apoptotic. Three independent experiments were performed. Representative results are presented.

### Immunoprecipitation

Expression plasmids bearing 2DEDplusE-FLAG or E protein-FLAG were transfected into Flp-in TREx 293 cells (Invitrogen) by using FuGene 6 Transfection Reagent (Roche). Six hours after tetracycline induction, both detached and attached cells were pooled, and cell extracts were prepared by washing the cells with PBS and adding 500 μl of lysis buffer (TrisHCl [pH 7.5], 150 mM NaCl, 1 mM EDTA, 1% Nonidet P20) containing Complete Protease Inhibitor (Roche). After a 30-min incubation on ice, the lysate was centrifuged at 15,000 rpm for 10 min in a microcentrifuge. Immunoprecipitation was performed according to a procedure described previously (9). Briefly, anti-FLAG M2 antibody and protein G sepharose were used. Aliquots of precipitate were analyzed by Western blotting using anti-FLAG antibody or anti-Caspase 8 (1C12) antibody (Cell Signaling Technology Inc.). The bands were detected using the ECLplus Western blotting detection system (Amersham Biosciences) according to the manufacturer's instructions and visualized with a Luminescent Image Analyzer LAS1000 (Fujifilm).

### Gel filtration of 2DEDplusE protein

Stable cell lines expressing 2DEDplusE-FLAG were plated onto four dishes at a density of 1 × 10^6^ cells per 100-mm dish with appropriate DMEM medium. Twenty-four hours after subculture, tetracycline was added. Six hours after tetracycline induction, cell extracts were prepared by washing the cells with PBS and adding 500 μl of Lysis buffer containing Complete Protease Inhibitor (Roche). After a 30-min incubation on ice, the lysate was centrifuged at 15,000 rpm for 10 min in a microcentrifuge. The supernatant was applied to a Sephacryl S-300 gel filtration column (Φ1.0 × 44 cm) equipped with a BIO-RAD Econo system, and size-fractionated fractions were collected. Aliquots of each fraction were subjected to electrophoresis and analyzed by Western blotting. Molecular weight marker proteins (Sigma) were applied to the same gel filtration column. The expression plasmid bearing E protein-FLAG was transfected into Flp-in TREx 293 cells (Invitrogen) using FuGene 6 Transfection Reagent (Roche). Six hours after tetracycline induction, cell extracts were prepared as described above.

## RESULTS

### Engineered DED of FADD produced a large assembled complex likened to an artificial death-inducing signaling complex (DISC)

**Figure 1:**
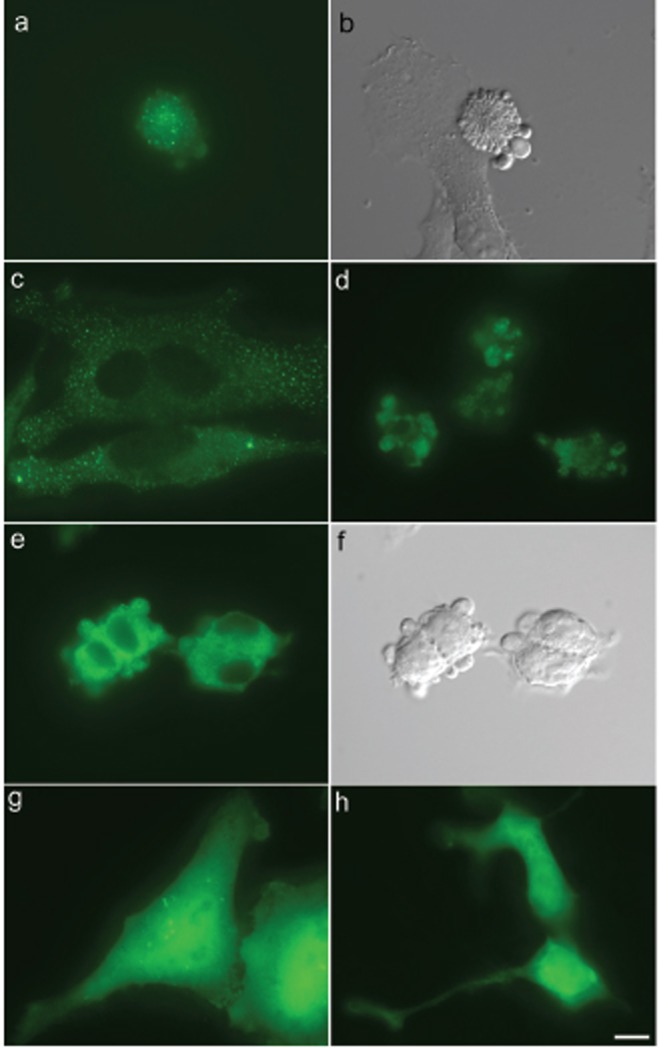
EGFP-tagged 2DEDplusE, an engineered FADD, forms artificial DISCs that appear as grain-like signals in the cytoplasm of apoptotic cells. (a), Epifluorescence micrograph of HeLa cells transiently expressing 2DEDplusE-EGFP showing the distribution of EGFP signal. Grain-like signals representing 2DEDplusE-EGFP were observed in apoptotic cells; (b), Same cells shown in (a) imaged by differential interference microscopy; (c), Epifluorescence micrograph showing the distribution of EGFP signal in non-apoptotic HeLa cells expressing 2DEDplusE-EGFP; (d), Epifluorescence micrograph of HEK293 cells transiently expressing 2DEDplusE-EGFP showing the distribution of EGFP signal; (e), HEK293 stable cell lines expressing 2DEDplusE-EGFP also displayed very small dots representing EGFP signal; (f), Same cells shown in (e) imaged by differential interference microscopy; (g), Epifluorescence micrograph showing the distribution of EGFP signal in HeLa cells transiently expressing E protein-EGFP; (h), Epifluorescence micrograph of HEK293 cells transiently expressing E protein-EGFP showing the diffuse cytoplasmic distribution of EGFP signal. Scale bar, 10 μm.

**Figure 2:**
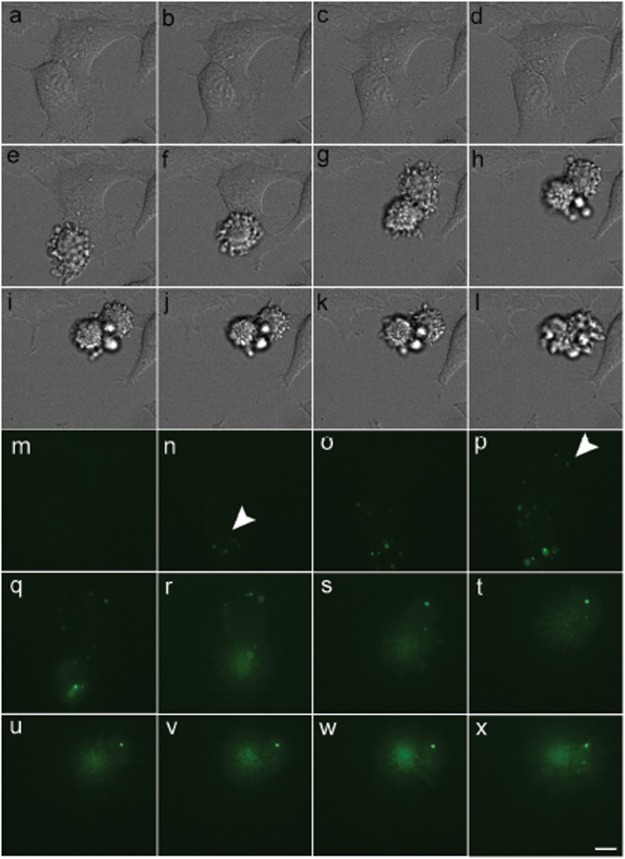
Membrane blebbing was observed shortly after the formation of grain-like signals. Serial time-lapse microscopy images showing the formation of grain-like signals and membrane blebs in HeLa cells transiently expressing 2DEDplusE-EGFP. Twenty-four hours after transient transfection, serial images of cells were taken at 10-min intervals. Images in e and g show the start of membrane blebbing. Arrowheads point to the first occurrences of grain-like signals in the cells. Scale bar, 10 μm.

To determine the state of 2DEDplusE in cells, we fused the C terminus of 2DEDplusE to EGFP to produce a 2DEDplusE-EGFP fusion protein. When HeLa cells were transfected with plasmids capable of producing 2DEDplusE-EGFP fusion protein, small grain-like signals were observed in apoptotic cells (Fig. 1a). In transfected HeLa cells that did not yet exhibit blebbing, many discrete small grain-like signals were observed in addition to signals representing weak staining that was uniformly distributed throughout the cytoplasm. The widespread distribution pattern of small grain-like signals (Fig. 1c) was common, but cells bearing such signals disappeared or changed in shape as apoptosis progressed. This phenomenon was confirmed in the serial images obtained through the time-lapse experiment (Fig. 2). Following the appearance of weak, uniformly distributed signals in cytoplasm, some signals became grain-like, probably due to the self-association activity of 2DEDplusE. Blebbing was observed approximately 30 min after grain-like signals were first detected (arrowheads in Fig. 2n, p). After blebbing, the strength of the EGFP signals appeared stronger and the number of EGFP signals increased.

Although HeLa cells displayed many small and discrete grain-like signals throughout their cytoplasm, HEK293 cells transiently expressing 2DEDplusE-EGFP displayed only a few grain-like signals. At least under the apoptotic conditions of our experimental system, HEK293 cells were more resistant to apoptosis than NIH3T3 and HeLa cells. The relationship between apoptosis resistance and low-number grain-like signals in HEK293 cells is interesting. To examine this relationship further, we established a stable cell line capable of producing 2DEDplusE-EGFP fusion protein under the control of the tetracycline repressor system. This stable cell line expressed 2DEDplusE-EGFP fusion protein at relatively low levels. After tetracycline induction, 2DEDplusE-EGFP signals appeared as very small dots in the cytoplasm (Fig. 1e). The apoptosis-inducing activity of 2DEDplusE-EGFP fusion protein in this stable cell line was lower than original 2DEDplusE, because EGFP occupation of the C terminus of 2DEDplusE may have impaired the fusion protein or may have caused the fusion protein to be unstable.

To test the state of E protein itself, we fused EGFP to the C terminus of E protein (this fusion protein was named Eonly-EGFP). In HeLa and HEK293 cells expressing only Eonly-EGFP, the signals were uniformly distributed throughout (Fig. 1g, h). In HeLa cells, a few dot-like signals were sometimes observed, but the majority of EGFP signals uniformly occupied the cell bodies. Expressing Eonly-EGFP in HeLa cells and HEK293 cells had no effect on apoptosis. Apoptosis was never induced. Although E protein was expected to self-associate in eukaryotic cells, fluorescence microscopy showed that most of the E protein in cells did not form large clusters. This does not necessarily mean, however, that E protein does not self-associate in cultured cells. It is well known that sufficiently high protein concentrations are necessary for self-assembling proteins to form large enough clusters that can be detected by fluorescence microscopy.

### Comparing the apoptosis-inducing activity of various engineered FADDs

**Figure 3:**
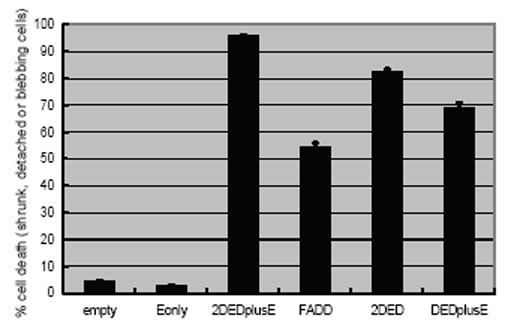
Cell death activity of various engineered FADD constructs. Cell death activity in HEK293 cells transiently expressing various constructs were assessed 24 hours after transfection and simultaneous tetracycline induction. Cell death activity was assessed by fluorescence microscopy. The basal level of cell death activity was determined by examining cells transfected with only pTREx-empty, a backbone vector.

We were interested in whether a single domain of DED and tandem domains of DED differ in their apoptosis-inducing activity. To investigate the effect of tandem DEDs, we assessed various engineered FADDs by quantitatively measuring apoptosis-inducing activity in HEK293 cells transiently transfected with tandem DED-containing expression plasmids. After transfection, expression was induced with tetracycline. Twenty-four hours after transfection, the percentage of apoptotic cells displaying membrane blebbing was counted. Cells transfected with empty vector were used as controls and to determine the cell toxicity of the transfection reagent, which was only 4% (Fig. 3). In cells expressing only E protein, virtually no apoptosis-inducing activity (2.8%) was observed. In cells expressing unmodified FADD, apoptosis-inducing activity was approximately 50%, whereas in cells expressing DEDplusE (single DED and E protein), apoptosis-inducing activity was approximately 70% (Fig. 3). Compared to the apoptosis-inducing activity in cells expressing DEDplusE, in cells expressing 2DEDplusE (tandem DEDs and E protein) apoptosis-inducing activity was approximately 95% (Fig. 3).

In these experiments, the apoptosis-inducing activity of 2DEDplusE was consistently stronger than that of DEDplusE. To our surprise, 2DED or tandem DED also exhibited strong apoptosis-inducing activity (82%), although slightly lower than that of 2DEDplusE. The enhanced apoptosis-inducing activity of 2DEDplusE was mainly due to the tandem DEDs. E protein also contributed to this enhanced activity but to a lesser extent. The apoptosis-inducing activity of DEDplusE was significantly greater than that of original FADD, indicating that E protein self-assembly contributed to the increased apoptosis-inducing activity of DEDplusE.

### 2DEDplusE protein can bind to caspase 8 without extrinsic signals

To investigate the mechanism underlying the apoptosis-inducing activity of 2DEDplusE, we fused a 3XFLAG tag to the C terminus of 2DEDplusE protein. HEK293 cells expressing 2DEDplusE-FLAG protein also underwent apoptosis, but 2DEDplusE-FLAG exhibited relatively slightly weaker apoptosis-inducing activity than original 2DEDplusE. Fluorescence microscopy of cells expressing 2DEDplusE-EGFP and cells expressing E protein-EGFP showed that 2DEDplusE-EGFP formed larger dot-like signals than E protein-EGFP, suggesting that tandem DEDs may also possess self-association activity. Alternatively, another cytoplasmic protein may bind the tandem DEDs to promote the assembly of 2DEDplusE-EGFP.

**Figure 4:**
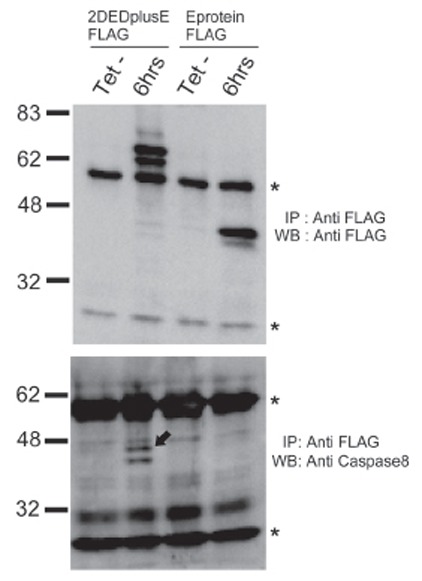
2DEDplusE binds procaspase 8 through DED domains. (A), Immunoprecipitation analysis was performed on extracts of HEK293 cells transiently transfected with C-terminal 3XFLAG-tagged 2DEDplusE or E protein. The analysis was performed 6 hours (lanes 2 and 4) after tetracycline induction with anti-FLAG antibody. Control experiments using extracts from cells cultured without tetracycline are shown in lanes 1 and 3. Precipitates were detected using anti-FLAG antibody and the ECLplus Western blotting detection system. Asterisks indicate heavy and light chains of IgG; (B), Aliquots of precipitates described for (A) were probed with anti-caspase 8 antibody. The arrow points to the p43/41 cleaved products of procaspase 8.

To investigate both of these possibilities, we sought to identify artificial DISCs and the type of proteins contained within the artificial DISCs. Either 2DEDplusE-FLAG or E protein-FLAG was transfected transiently into HEK293 cells, and 6 hours after tetracycline induction, extracts from these cells were used for immunoprecipitation using anti-FLAG antibody (Fig. 4). The precipitates were then analyzed by Western blotting with anti-caspase 8 antibody. The weak but discrete p43/p41 signals of caspase 8 were detected only in extracts derived from 2DEDplusE-FLAG-expressing cells. Full procaspase 8 was not detected because its size overlapped with the heavy chain of anti-FLAG antibody. We surmise, however, that the amount of full procaspase 8 in the 2DEDplusE-FLAG complex was very low, because 6 hours after tetracycline induction full caspase 8 was rarely detected, even in the original whole-cell extracts that were not subjected to immunoprecipitation (data not shown). This clearly showed that 2DEDplusE bound partially cleaved caspase 8. Most likely in the first step, full-length procaspase 8 bound tandem DED, then the procaspase 8 molecules cleaved each other. If caspase 8 cleavage should progress, the mature forms of caspase 8, p18 and p10, should be released from the 2DEDplusE complex into the cytoplasm. The 2DEDplusE complex significantly increased the concentration of procaspase 8 specifically in the cytoplasm, promoted caspase 8 cleavage by positioning caspase 8 molecules so that they could cleave each other, and finally strongly activated caspase 8. This complex, therefore, represents an artificial DISC composed of 2DEDplusE.

To determine what type of proteins the artificial DISC of 2DEDplusE contains, we analyzed cFLIP and FADD by immunoprecipitation and Western blotting with specific cFLIP and FADD antibodies. However, cFLIP and FADD was not detected. We concluded that the artificial DISC of 2DEDplusE does not contain enough cFLIP and FADD to be detected by immunoprecipitation and Western blotting.

### 2DEDplusE forms a large complex containing partially cleaved procaspase 8 (p43/p41)

**Figure 5:**
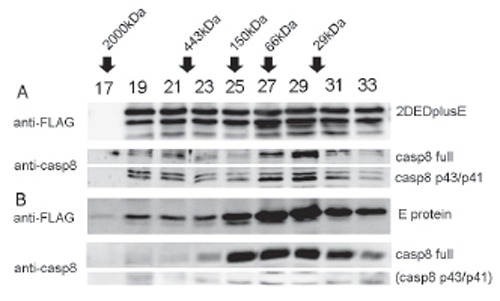
Identification of 2DEDplusE complexes through gel filtration analysis. HEK293 cells transiently transfected with C-terminal 3XFLAG-tagged 2DEDplusE (A) or E protein (B) were analyzed. Six hours after tetracycline induction, cells extracts were prepared and then subjected to Sephacryl S-300 gel filtration chromatography. Aliquots of eluates were analyzed by Western blotting with anti-FLAG or anti-caspase 8 antibodies. Calibration was performed using the following molecular-weight markers: blue dextran 2000 (2000 kDa), apoferritin (443 kDa), alcohol dehydrogenase (150 kDa), bovine serum albumin (66 kDa), and carbonic anhydrate (29 kDa).

To examine whether an artificial DISC of 2DEDplusE is present in cells and to exclude the possibility that 2DEDplusE binds p43/p41 of caspase 8 as a result of the immunoprecipitation manipulation, we isolated the complex from cells stably expressing 2DEDplusE-FLAG. Six hours after tetracycline induction, cell extracts were prepared and subjected to gel filtration chromatography. The molecular weight of the complex was determined by Sephacryl S-300 gel filtration. After gel filtration, we analyzed aliquots of each fraction by Western blot using anti-FLAG and anti-caspase 8 antibodies (Fig. 5). The 2DEDplusE-FLAG peak distributed broadly to fractions 19–33. This broad gel filtration peak indicates that various sizes of the complex were present. The 2DEDplusE-FLAG signals found in fractions 19–21 clearly demonstrated that 2DEDplusE (approximately 60 kDa) formed a large complex greater than 443 kDa, the molecular weight of the apoferritin calibration marker protein. The peaks representing 2DEDplusE-FLAG and E protein-FLAG eluted from gel filtration chromatography remained in the low molecular-weight fraction. This might result from interactions between the proteins and Sephacryl resin or from overload of protein samples. Compared to E protein-FLAG eluates, a significant proportion of 2DEDplusE-FLAG eluates shifted to high molecular-weight fractions. Western blotting of fractions 19–21 with anti-caspase 8 antibody revealed that the high molecular-weight fractions from which 2DEDplusE-FLAG complex was eluted also contained p43/p41 of caspase 8. On the basis of the distribution of the high and low molecular-weight fractions, we determined that approximately one-third of caspase 8 existed in the complex form in cells expressing 2DEDplusE-FLAG.

E protein is known to self-assemble in *E. coli* infected with lambda phage (4, 5). To determine whether E protein also self-assembles in eukaryotic cells, we examined whether E protein-FLAG forms self-association complexes in HEK293 cells expressing E protein-FLAG. To detect E protein-FLAG complexes, we subjected cell extracts containing E protein-FLAG to Sephacryl S-300 gel filtration chromatography. The resulting eluate was analyzed by Western blot using anti-FLAG antibody. Most E protein-FLAG eluted around fractions 27–29, which is consistent with a monomer of 44 kDa. In addition to the monomer, a secondary peak having a weak but significant signal was also observed in fraction 19, a fraction containing higher molecular weight (over 2000 kDa) compounds, clearly indicating that a population of E protein-FLAG is capable of self-assembling under eukaryotic cell conditions. Contrary to our expectations, most E protein-FLAG was present in monomer form. This might be related to the cytoplasmic conditions of eukaryotic cells, the effect of the FLAG tag on the C terminus of E protein, or to the relatively lower concentration of protein produced in eukaryotic cells than in *E. coli*. Generally, protein oligomerization depends on protein concentration (10).

Although Western blotting of extracts from E protein-FLAG-expressing cells detected full caspase 8, it did not detect cleaved caspase 8. Moreover, when these extracts were subjected to gel filtration chromatography, neither full nor cleaved caspase 8 eluted to the large molecular-weight fraction.

To confirm that pure E protein self-associates, we fused the N terminus of E protein and 2DEDplusE to GST protein and expressed the fusion proteins in *E. coli*. After purification through a standard glutathione-sepharose 4B procedure, pure proteins were subjected to Sephacryl-S300 gel filtration chromatography. The main GST-2DEDplusE peak occurred around fraction 19; GST-E protein eluted from the void fraction to fractions 17–21 with long tails (data not shown). Although GST is a dimeric enzyme composed of 26-kDa monomers, these gel filtration chromatography results on pure E protein and 2DEDplusE indicated clearly that these proteins are able to assemble without the aid of other proteins.

## DISCUSSION

The engineered FADD made in our study greatly enhanced apoptosis-inducing activity in adherent cells. The tandem DED of 2DEDplusE contributed most to the enhancement of apoptosis and the E protein contributed less so. Membrane blebbing associated with apoptosis was observed just after formation of grain-like signals. Furthermore, 2DEDplusE can bind p43/p41 forms of caspase 8 but E protein cannot, indicating that 2DED-bound procaspase 8 activates procaspase 8. In the absence of an extrinsic signal, the engineered FADD forms artificial DISC in the cytoplasm, then its tandem DED activates procaspase 8, which in turn executes apoptosis. Thus, we showed that engineered FADD complex closely mimics intrinsic DISC and increases apoptosis-inducing activity.

Within the DISC (11), each DD of activated Fas receptor trimers binds one molecule of FADD through homotypic interactions between the DDs (6). Biochemical analysis reveals that self-association between the DEDs of FADD molecules is also important for DISC assembly (12). From their study of the structure of FADD, Carrington *et al*. proposed that DISC is structured such that Fas receptor, FADD, and procaspase 8 organize into a hexagonal lattice or honeycomb-like structure that extends the death receptor array after receptor activation (13). Although FADD and procaspase 8 are constitutively expressed in cells, in the single interaction state the affinity between FADD and Fas receptor or interactions between FADD and FADD are believed to be relatively low (13). In the “on” state, however, these weak interactions between FADD and Fas and between FADD and itself work cooperatively, causing them to cluster into higher order complexes in a FADD-dependent manner and to form a cluster of activated, membrane-localized receptors such as DISC (13).

We expected the mechanism of action of the engineered FADD-2DEDplusE-to mainly involve the E protein due to its self-assembly activity. We did not expect the tandem DEDs to enhance apoptosis-inducing activity. These ideas were consistent with our previous findings that 2DED2DD, an engineered FADD produced by fusing tandem DEDs and tandem DDs, displays moderate apoptosis-inducing activity (3). In stably expressed cell lines, the apoptosis-inducing activity of 2DED2DD is stronger than that of unmodified FADD but weaker than that of 2DEDplusE (3). In the present study, however, only 2DED promoted early-stage apoptosis blebbing or cell shrinkage in human HEK293 cells that transiently expressed 2DED by means of a tetracycline-inducible TREx system. The TREx expression system can produce foreign proteins at an extremely high level in most transfected cells, but foreign protein expression levels actually vary in each cell due to transient transfection. It is still unknown whether, at low expression levels, only 2DED can execute apoptosis. Since E protein in 2DEDplusE certainly enhanced the apoptosis-inducing activity of 2DED (Fig. 3), we speculate that, at low protein levels, E protein may also effectively enhance apoptosis-inducing activity.

We also concluded that 2DED mimicked the dimerization that occurs through the self-association of DED in FADD, and that E protein mimicked the multimerization that occurs through the interaction between DD in activated Fas receptor trimers and FADD. The DED of 2DED still has the ability to bind the other DED of 2DED and the DED of intrinsic FADD. This ability to self-associate also contributes to the enhancement of apoptosis-inducing activity. We believe that this complex represents an artificial DISC or mimics DISC for three reasons. Firstly, 2DEDplusE forms large protein complexes. The cytoplasm of cells expressing 2DEDplusE-EGFP was observed to contain dot-shaped signals visible under fluorescence microscopy. Moreover, gel filtration analysis demonstrated that 2DEDplusE-FLAG eluted to high molecular-weight fractions (14). Because 2DEDplusE complex eluted to a wide range of fractions, from a fraction corresponding to molecular weights of over ∼2,000 kDa to a fraction as low as 71 kDa (monomer size), various sizes of 2DEDplusE complex exist in the cytoplasm. Secondly, the high molecular-weight fraction containing the 2DEDplusE complex contained p43/p41 forms of caspase 8, as demonstrated by Western blot analysis. Immunoprecipitation analysis also demonstrated that 2DEDplusE-FLAG bound p43/p41 forms of caspase 8. These results clearly show that the 2DEDplusE complex also contains caspase 8, which is also a component of intrinsic DISC (15).

Finally, the form of caspase 8 contained with the 2DEDplusE complex is the p43/p41 intermediate form of activated caspase 8, not full-length procaspase 8. As the first processing step, p43/p41 caspase 8 is processed by caspase 8. The presence of p43/p41 caspase 8 in the 2DEDplusE complex supports the premise that procaspase 8 molecules within the complex can activate and process each other (6). Moreover, in cells expressing 2DEDplusE, procaspase 8 was processed to p43/p41, whereas in cells expressing only E protein, procaspase 8 was not processed to p43/p41. These results strongly suggest that 2DEDplusE protein oligomers bind procaspase 8 through DED-DED interactions, and then accumulated procaspase 8 undergoes autoproteolytic processing, which similarly occurs during the activation of intrinsic DISC.

Apoptotic defects resulting from epigenetic alterations and loss of heterozygosity are believed to be responsible for tumor formation, progression, and resistance to anti-cancer drugs (16). Various types of cancer cells attempt to escape the apoptosis cascade through different strategies. Our aim is to develop a reliable means of inducing the complete apoptosis cascade in a given targeted cell type. In line with this aim, we continue to develop other types of engineered apoptotic factors. In this paper, we report that tandem DED and 2DEDplusE have potent apoptotic effects that ultimately induced artificial DISC formation in the cytoplasm without extrinsic signals. These findings provide valuable information that will aid the future development of a more potent apoptosis-inducing factor.
